# Safe implementation of minimally invasive surgery in a specialized colorectal cancer unit

**DOI:** 10.1007/s10151-024-03019-w

**Published:** 2024-11-16

**Authors:** José Azevedo, Anna Kashpor, Laura Fernandez, Ignacio Herrando, Pedro Vieira, Hugo Domingos, Carlos Carvalho, Richard Heald, Amjad Parvaiz

**Affiliations:** 1 Digestive Unit, Champalimaud Clinical Center, Champalimaud Foundation, Lisbon, Portugal; 2 Faculty of Medicine, University of Lisbon, Lisbon, Portugal; 3 Biophotonic Laboratory, Champalimaud Research, Champalimaud Foundation, Lisbon, Portugal; 4 University of Portsmouth, Portsmouth, UK

**Keywords:** Colon cancer, Rectal cancer, Minimally invasive surgery, Robotic surgery, Laparoscopic surgery: surgical training

## Abstract

**Introduction:**

In the past 30 years, minimally invasive surgery (MIS) has made remarkable progress and has become the standard of care in colorectal cancer treatment. The implementation of new techniques or platforms is, therefore, a challenge for surgical teams. This study aims to analyze the experience in the implementation of minimally invasive surgery in the colorectal unit in a specialized colorectal cancer center. We will report and compare the clinical outcomes of the patients submitted to the different surgical approaches, reflecting the importance of surgical training in the laparoscopic and robotic field for the reduction of surgical complications and improve short-term outcomes.

**Methods:**

This study involved a retrospective analysis of data collected from a prospectively maintained database at the colorectal unit of Champalimaud Foundation between 2012 and 2023. Data were collected as part of routine clinical documentation and included variables on patient’s demographics, staging, short-term outcomes, and follow-up.

**Results:**

A total of 661 patients treated at the Champalimaud Foundation between 2012 and 2023 were included, of which 389 (59%) had colon and 272 (41%) rectal cancer. Most of the patients underwent elective surgery, with a minimally invasive approach performed in 91% of cases. A complete resection (R0) was achieved in 95.1% (619) of the procedures with a pathology report staging 64.5% (409) of tumors as pT3–4. Eleven percent (70) of patients had complications classified as Clavien-Dindo (CD) ≥ 3.

**Conclusion:**

This study supports the safety of the implementation of minimally invasive surgery in colorectal cancer care, with improvement in postoperative outcomes and surgical quality, supporting the importance of surgical training and specialized teams.

## Introduction

### Colorectal surgery

In the last 30 years, minimally invasive surgery (MIS) has made remarkable progress [1] and has become the standard of care of colorectal cancer treatment. Since the first report on laparoscopic resection in 1991 [2], several randomized controlled trials (RCTs) have shown its non-inferiority or equivalence compared to open surgery in colon cancer. However, the results have been more controversial for rectal cancer, leading to a delay in implementation of MIS in this subgroup of patients [3] and to the increase of alternative MIS techniques such as transanal total mesorectal excision (TaTME). Nonetheless, in rectal cancer, robotic surgery has been growing in popularity since 2002. Robotic techniques are characterized by operator-controlled, three-dimensional, high-resolution vision and include a stable camera platform and robotic arms with a wide range of motion and tremor elimination. Thus, they offer a more detailed view of the surgical field and similar or even better flexibility than a surgeon’s hand, allowing more accurate and highly precise surgery [4, 5]. The transition from conventional surgery to minimally invasive surgery may imply a vulnerable process with a necessary learning curve, which may jeopardize patient outcomes.

Both oncological and functional outcomes after surgery are factors where the skill of the operating surgeon has a major impact [6]. Rectal cancer surgery imposes important challenges for the surgical teams. First, due to the confines of the pelvis, limitations of exposure, risk of injury to adjacent structures, and need for extended procedures such as abdominoperineal resection, about 25% of patients may require higher technical expertise [2]. Second, the introduction of neoadjuvant chemoradiotherapy or radiotherapy has posed some additional challenges, as irradiated tissue may be more difficult to manage surgically. Third, rectal cancer surgery can cause many functional consequences affecting quality of life of survivors.

The implementation of new techniques or platforms is, therefore, a challenge for surgical teams. Unregulated adoption of novel surgical techniques may lead to poor results; as such, a structured training program should be an essential component for any department or team that wants to train on new techniques or platforms. A training program in minimally invasive surgery should include permanent evaluations of surgeons and use of techniques such as simulation, telementoring, proctoring, and evaluation of surgical videos by experts.

The Digestive Cancer Multidisciplinary Unit of the Champalimaud Clinical Centre was created in late 2013. The colorectal cancer team has developed dynamic work that includes implementation of minimally invasive approaches such as laparoscopy and robotic surgery. During this period, a need for training surgeons from outside the institution was recognized. As such, seeing such a need for robotic colorectal surgery, the European Academy of Robotic Colorectal Surgery (EARCS) was founded at our institution. EARCS provides a framework and guidelines for selecting appropriate surgeons, skill courses, and direct supervision of clinical cases for robotic colorectal surgery. EARCS is audited by collecting clinical results, while surgical performance is evaluated using a structured process for formative and summative assessment. Since October 2014, more than 150 surgeons have registered with the training program and at least 80 surgeons from 54 centers in 15 European countries have graduated from the academy [7]. The methodology of this program was previously published [8].

As part of the constant auditing of clinical results, this study aims to analyze the experience of implementation of minimally invasive surgery in a colorectal unit from a specialized cancer center in terms of safety and impact in short-term clinical outcomes. We aim to report and compare the short-term outcomes of the patients submitted to the different surgical approaches and their evolution along time.

## Methodology

### Data collection

This study includes a retrospective analysis of data collected from a prospectively maintained database at the colorectal unit of Champalimaud Foundation. The database contains information on all consecutive colorectal resections performed at the unit, for primary cancer. The unit was officially created in 2013; however, one patient underwent surgery in 2012. To avoid excluding any of our patients from the study, we decided to include the patient operated on in 2012. Data were collected as part of routine clinical documentation and encompassed variables on patient’s demographics, staging, and short-term outcomes. Local ethical approval and informed consent were obtained.

Demographic variables included age, gender, BMI (body mass index), ASA classification (I–IV), type of surgery (elective, emergent, urgent), surgical approach (open, laparoscopic, robotic, and transanal), and the characteristics of colonic and rectal resections (type of tumor, previous treatment, surgical procedure, stoma, retrieved lymph nodes, complete resection rate, conversion rate, and pathology report). Complications encompassed type of resections, anastomotic leak, reoperation, and reintervention rate. Surgical complications were classified according to Clavien-Dindo.

### Statistical analysis

Data analysis was conducted using STATA/C 14.2 for Mac (64-bit Intel), revised 16 March 2017, Copyright 1985–2015 StataCorp LLC. Non-parametric data were expressed as median with interquartile range and parametric data as mean with standard deviation. An analysis of type of approach over time was conducted to understand the evolution of the best approach in the department. A multivariate logistic regression analysis was performed to assess whether date of operation and baseline characteristics affected short-term outcomes while MIS implementation was occurring. Variables with *p* < 0.05 were considered statisticaly significant. The constant was included in the analysis model, and data are presented as odds ratio, 95% confidence interval, and *p* value.

## Results

This study included 661 patients treated at Champalimaud Foundation between 2012 and 2023. Of the patients, 307 (46%) were female, and 389 (58.9%) received colon and 272 (41.1%) rectal surgery. The median age was 65 years; median BMI was 26 kg/m^2^. A total of 488 (74.6%) patients were ASA I or II, and most tumors were staged as T3 (274, 56%), cN positive (241, 52%). Concerning rectal cancer, the median distance from the anal verge was 7.9 cm, and patients received neoadjuvant radiotherapy in 47% (133) of cases. Detailed patient characteristics are given in Table 1.

**Table 1:** Patient and tumor characteristics; Univariate analysis for CD>=3.

Patient and tumor characteristics (percentage of missing data)	Total (*N* = 661)	*p*-value
Age, median (IQR) *(1)*	65 (22–92)	0.932
Female, *(0)*	307 (46%)	0.157
BMI, median (IQR) *(11)*	26 (17–43)	0.007
ASA, *(1)*		0.852
I–II	488 (74.6%)	
III–IV	166 (25.4%)	
Location, *(0)*		0.592
Colon	389 (58.9%)	
Rectum	272 (41.1%)	
Distance from anal verge, median (IQR)	7.9 (5–10)	0.691
Clinical staging		0.768
cT *(26)*		
T1	41(8.4%)	
T2	103 (21.2%)	
T3	274 (56.3%)	
T4	69 (14.2%)	
cN *(30)*		
N0	217 (47.3%)	0.45
N +	241 (52%)	
EMVI ( +) *(24)*	66 (31.4%)	0.995
CRM ( +) *(0)*	76 (25.7%)	0.537
Previous treatment *(0)*		0.506
Radiation	133 (47.7%)	
Short course	41 (30.8%)	
Long course	92 (69.2%)	
TNT	88 (34.6%)	

### Surgical treatment

The most performed procedure was a total mesorectal excision in 202 (30.7%) patients followed by right hemicolectomy in 173 (26.6%) and sigmoidectomy in 123 (18.9%). Sixty-two (9.5%) patients received an abdominoperineal resection. Three hundred twenty-six (49.3,%) patients were treated laparoscopicaly, 268 (41%) robotically, and 60 (9.0%) by open approach. There were 22 conversions (3.6%), 20 (3.3%) in laparoscopy and 2 (< 1%) in robotic surgery. A median of 24 lymph nodes was reported in each specimen, and the mesorectum was intact in 87.7% (228) of rectal cancer surgery patients. An R0 resection was achieved in 95.1% (619) of the procedures with a pathology report staging 64.5% (409) of tumors as pT3-4. Treatment characteristics are shown in Table 2.

**Table 2:** Surgical treatment and short-term outcomes; Univariate analysis for CD>=3.

Surgical treatment and short-term outcomes (percentage of missing data)	*N* (%)	*p*-value
Surgical procedure		0.012
Ileocecal resection	98 (1.2%)	
Right hemicolectomy	184 (27.9%)	
Transverse resection	7 (1.1%)	
Left hemicolectomy	50 (7.6%)	
Sigmoidectomy	124 (18.8%)	
Low anterior resection	202 (30.7%)	
Abdominoperineal resection	58 (8.8%)	
Hartmann	4 (0.6%)	
Transanal	7 (1.1%)	
Subtotal colectomy	7 (1.1%)	
Panproctocolectomy	3 (0.5%)	
Pelvic exenteration	5 (0.8%)	
Surgical approach, (0)		0.48
Open	60 (9.08%)	
Laparoscopic	326 (49.3%)	
Robotic	268 (40.5%)	
Transanal	7 (1.1%)	
Minimally invasive surgery, *(0)*		0.839
Yes	601 (90.9%)	
Conversion to open, *(0)*		0.019
Total	22 (3.6%)	
Laparoscopic	20 (3.3%)	
Robotic	2 (< 1%)	
Stoma, *(3)*	238 (37.2%)	0.152
Oncological pathology report		0.431
pT (3)		
T0	36 (5.7%)	
Tis	4 (0.6%)	
T1–2	185 (29.2%)	
T3–4	409 (64.5%)	
N + (3)	220 (36.5%)	0.201
Retrieved lymph nodes, median (IQR)	24 (0–74)	0.894
Mesorrectal quality *(0)*		0.606
Mesorrectal plane	228 (87.7%)	
Intramesorectal plane	20 (7.7%)	
Muscular plane	12 (4.6%)	
Complete resection* (0)*		0.274
R0	619 (95.1%)	
R1	27 (4.2%)	
R2	5 (0.8%)	
Anastomotic leak, *(0)*	23 (4.2%)	–
Postoperative complications (Clavien-Dindo) *(0)*		–
1	37 (5.6%)	
2	108 (16.3%)	
3		
3a	17 (2.5%)	
3b	47 (7.1%)	
4		
4a	5 (0.8%)	
5	1 (0.2%)	
Clavien-Dindo ≥ 3,	70 (11%)	–
Length of stay, median (IQR) *(11)*	6.7 (1–26)	

### Implementation of minimally invasive surgery

**Fig. 1:**
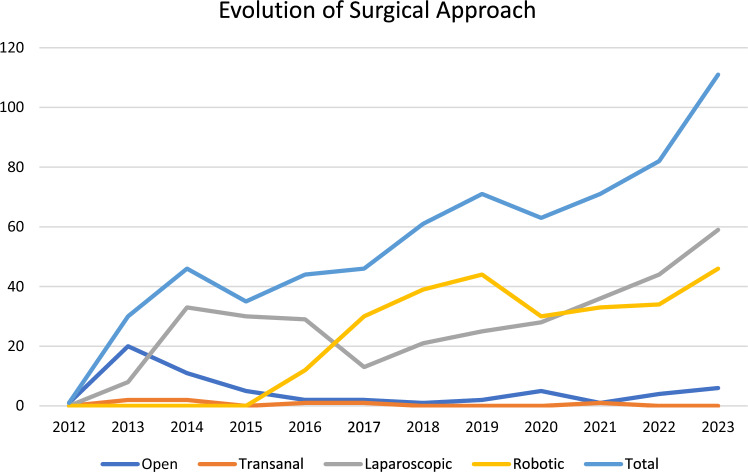
Evolution of approach and number of resections from 2012 to 2023

As seen in Fig. 1, there was an increase in the number of resections from 2012 to 2023. This was in accordance with the development of the colorectal surgical unit. During the first 2 years, there was an increase in the use of laparoscopy (2012 to 2014). Subsequently, in the following year, 2015, the robotic platform was started by the colorectal team. The number of patients treated with open approach diminished as soon as laparoscopy was introduced and plateaued at a low number (< 5 per year) after the introduction of the robotic system.

### Surgical complications

Seventy (11%) complications with Clavien-Dindo ≥ 3 were seen in the entire period. There was a slight increase in the absolute number of CD ≥ 3 complications from 2013 to 2023, with 4 and 12 complications, respectively. However, the ratio between complications with CD ≥ 3 and the number of procedures was lower in 2023 (11%) than at the beginning of the study in 2013 (13%). Details of complications per year and type of approach are shown in Table 3.

**Table 3:** Description of number of cases performed and complications during the study period.

	Open	Transanal	Laparoscopic	Robotic	Total procedures	CD ≥ 3	CD ≥ 3/procedures (%)
2012	1	0	0	0	1	0	0
2013	20	2	8	0	30	4	13
2014	11	2	33	0	46	0	0
2015	5	0	30	0	35	3	9
2016	2	1	29	12	44	5	11
2017	2	1	13	30	46	5	11
2018	1	0	21	39	61	6	10
2019	2	0	25	44	71	9	13
2020	5	0	28	30	63	8	13
2021	1	1	36	33	71	8	11
2022	4	0	44	34	82	10	12
2023	6	0	59	46	111	12	11
Total	60	7	326	268	661	70	11

**Fig. 2:**
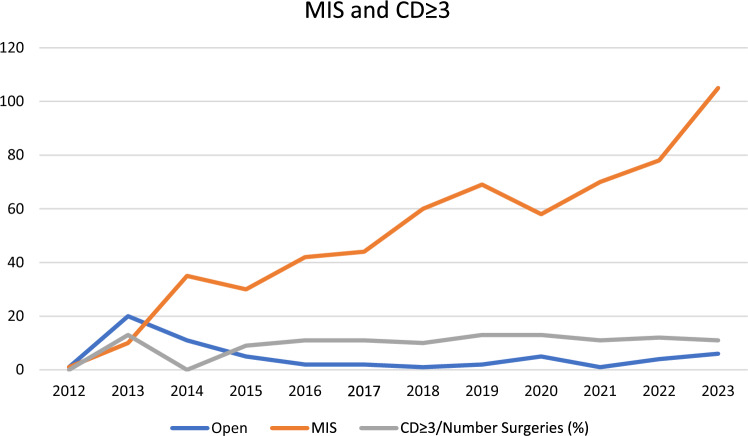
Number of CD ≥ 3 complications and use of the minimally invasive (MIS) approach from 2012 to 2023

During 2013, patients were mainly treated using an open approach, with a ratio CD ≥ 3/procedures of 13%. There was an increase in patients treated per year with MIS and an increase in the total number of patients treated per year, as shown in Fig. 2. In 2023, there was an increase in use of the robotic platform and more than double the number of patients treated (111) by the surgical team compared to 2016 when the robotic program started. However, during this time period, the ratio of CD ≥ 3/procedures since 2016 plateaued after introduction of robotic platform to close to 10% per year.

Type of surgical procedure, BMI, and conversion to open procedure were correlated with having a CD ≥ 3 complication in univariate analysis. However, after multivariate regression, no risk factor in our cohort was independently associated with having a CD ≥ 3 complication. Details are given in Table 4.

**Table 4:** Multivariate logistic regression for CD ≥ 3

	Odds ratio	SE	*p*-value	95% confidence interval]	
BMI	1.055226	0.033695	0.092	0.9912091	1.123377
Type of procedure	1.039605	0.072557	0.578	0.9066933	1.191999
Conversion (MIS)	1.942235	1.292508	0.319	0.5270465	7.157389

## Discussion

Colorectal surgery has had a notable shift from traditional open surgery to minimally invasive techniques over recent years. Initially, laparoscopic surgery gained prominence as a less invasive alternative, demonstrating clear benefits over open surgery, including faster return of bowel function, less postoperative pain and opioid use, and shorter length of stay. However, a trend toward adopting robotic-assisted surgery emerged, providing high-resolution three-dimensional imaging and superior ergonomics, with potential to overcome technical limitations of laparoscopy.

In this study, there was a pronounced preference for minimally invasive surgical approaches (91%), reflecting the current standard in colorectal cancer management. Notably, laparoscopic procedures were more frequently utilized for colonic interventions, whereas robotic-assisted surgeries predominated in rectal cases. This distribution aligns with the prevailing literature, where the most comprehensive studies on robotics in colorectal cancer have specifically emphasized its application in rectal cancer treatment [9, 10].

At the beginning of the study period, there was an increase in the use of laparoscopy, which was progressively substituted by an increase in robotic surgery. At the same time, as expected, a decrease in the proportion of open surgery was observed in the first years of the study period. The biggest concern when transitioning from open approach to MIS is the increase of intra- and postoperative complications during the learning period. Our study found that during the implementation of both laparoscopic and robotic systems, the increase in CD ≥ 3 complications did not change. As a matter of fact, at the end of the study period (2023) twice the number of operations were performed by the surgical team with the same absolute value of complications.

We believe that the main reason for these results is the application of modular, systematized, and permanently audited learning. This method is performed by the most experienced senior surgeon and has been advocated as the method of choice when learning advanced surgical techniques, having no deleterious effects on clinical outcomes. This goal-oriented, targeted teaching method enables a single experienced surgeon to prepare several senior colorectal surgeons for independent MIS practice at the same time. A major benefit of modular training is its applicability across a wide spectrum of patients. With this module, it is possible to provide at least one module of training during 98% of all colorectal resections even though most patients have adverse factors regarding conversion/technical difficulty [11].

### Surgical quality

Total mesorectal excision completeness has become a marker for good surgical technique in rectal cancer and predicts the likelihood of local recurrence of the cancer in the pelvis [12]. In the population presented, surgery was performed according to the mesorrectal plane in 87% of patients, which is comparable to the results observed in ACOSOG and COLOR II Trial (85%) and higher than those in the ROLLAR (75%) and COREAN Trials (73.6%) [10, 13–15]. Although this rate is lower than in the REAL Trial (93%) [9], there are patients submitted to laparoscopic and open treatment in our population and a higher rate of lower tumors, which may explain this difference.

Concurrent with the high quality of mesorectal excision, a high rate of R0 resections was observed in our study (95%), suggesting that patients in our center receive effective treatment regarding complete tumor removal with clear margins and minimal risk of recurrence, aligning closely with the standards established by several widely recognized RCTs in the colorectal field.

These results collectively provide evidence showing the efficacy and success of the implementation of MIS in colorectal cancer in this center regarding oncological outcomes.

### Conversion rate

The conversion rate in our study was significantly lower compared to the literature (11–25%) [14, 16–18]. According to the CLASICC Trial, the most common causes for conversion in colon cancer were excessive tumor fixity, uncertainty of tumor clearance, and obesity, while in the COST Trial they were advanced disease, no visualization of critical structures, and adhesions [17]. As a tertiary center, we managed to treat patients with more advanced disease, maintaining a low conversion rate. Notably, in our cohort of rectal cancer patients, conversion occurred only in mid-rectal cancers. In the COLOR II trial, mid-rectal cancers also had the highest risk of conversion [14]. Although the explanation is still not clear, it is known that in laparoscopy there is a limited of angle of operation that can make the resection of middle and low rectal cancer difficult.

Concerning the robotic approach, in rectal cancer there were no conversions to open surgery, which aligns with the advantages of robotic surgery in rectal cancer described in the literature [7–10]. Globally, the conversion rate was higher in laparoscopic compared to robotic surgery in both colon and rectal cancers, similar to results provided in the ROLARR and REAL Trials [9, 10].

The overall low conversion rate in this study highlights not only the effective approach taken towards MIS in this center but also the high level of safety of these procedures. Conversion to open surgery is often used to assess the MIS learning curve, as conversion rate is mainly associated with the experience of the surgeon, hospital volume, and case complexity.

### Short-term outcomes

Our results demonstrate a 32% total number of complications. This overall rate is lower than the literature rate of 40–58% [13, 14, 18]. Similarly, in our study, severe complications (Clavien-Dindo ≥ 3) occurred in only 11% of all colorectal cancer resections, which is lower than the rate in the available literature [13, 19] when compared to well established units.

The anastomotic leak rate was 4.2%, which is lower compared to 8.5% inthe CLASICC Trial, 12% in the COLOR II Trial, and 11% in the ROLAR Trial [10, 14, 18]. Several factors can contribute to the risk of anastomotic leak [20], namely patient’s comorbidities, preoperative chemotherapy, type of anastomosis, tumor stage and location, use of protective stoma, surgeon’s experience, and postoperative care, although some of these are still controversial in the literature.

Regarding the length of stay (LOS) after surgery, the previously cited studies indicate a mean of 5 to 13 days, whereas the median hospital stay is 6 days. However, the hospital stay may be influenced not only by time to recovery and quality of postoperative care but also by social factors such as differences in medical fees, medical insurance, and medical systems among countries [21].

The low rate of postoperative complications, mainly severe complications (CD ≥ 3) and anastomotic leak, is a positive indicator of quality and safety, demonstrating that there can be a shift from open to MIS with minimal risks to the patient. Furthermore, the short LOS suggests faster recovery and reduced risk of hospital-acquired complications, supporting that the implementation of MIS in colorectal cancer is not only safe but also efficient and has socioeconomical advantages over open surgery.

The future directions of MIS will bring the integration of artificial intelligence (AI), which is expected to provide real-time decision support and predictive analyses during surgery. In addition, robotic platforms are expanding to allow for more complex procedures, which could lead to greater use in advanced and recurrent colorectal cancer. These advances should be designed to provide better access training and standardization as well as cost-reduction to facilitate generizability.

### Limitations and strong points

One of the primary strengths of this study is its external validity owing to the utilization of population-based data that mirror daily clinical practice. This ensures that the findings are widely applicable and reflective of real-world scenarios. Additionally, the integrity of the dataset is supported by the minimal number of missing data, which contributes to the robustness and reliability of the results presented. Due to the minimal number of missing data concerning our main outcomes, a list-wise deletion approach was preferred.

Limitations of this study included that this was a single-institution retrospective analysis, with inherent selection bias. There is a limited generalizability to other healthcare settings, as patient populations and surgical practices can vary between institutions. Furthermore, this study primarily focuses on short-term outcomes and perioperative measures. Long-term oncological outcomes and patient survival data may not be available or sufficiently analyzed in this study, which limits the ability to draw conclusions about the long-term effectiveness of MIS.

Despite these potential drawbacks, it is reassuring that our results are similar to those previously published in the more representative RCTs in this field.

## Conclusion and future prospects

The gradual paradigm shift that occurred from open surgery to MIS was not associated with an increase in postoperative complication rates or a decline in surgical quality and short-term clinical outcomes compared to widely recognized RCTs that proved the safety of laparoscopic and colorectal surgery in the last decades. In our study, there was a high rate of R0 resections and high-quality surgery, indicating effectiveness of MIS in oncological resections, along with a low conversion rate and postoperative complication rate, supporting the safety of the implementation of MIS in colorectal cancer.

The continued integration of MIS into colorectal surgery is promising, and there is a compelling rationale for its continued adoption in clinical practice. Future research should explore long-term oncological outcomes and patient-reported quality of life to provide a comprehensive assessment of the benefits of these approaches.

## Data Availability

No datasets were generated or analysed during the current study.

## References

[1] Morneau M, Boulanger J, Charlebois P, Latulippe JF, Lougnarath R, Thibault C, Gervais N (2013) Laparoscopic versus open surgery for the treatment of colorectal cancer: a literature review and recommendations from the Comité de l’évolution des pratiques en oncologie. Can J Surg 56(5):297–310. 10.1503/cjs.005512 24067514 10.1503/cjs.005512 PMC3788008

[2] Jacobs M, Verdeja JC, Goldstein HS (1991) Minimally invasive colon resection (laparoscopic colectomy). Surg Laparosc Endosc 1:144–150 1688289

[3] Yamauchi S, Matsuyama T, Tokunaga M, Kinugasa Y (2021) Minimally invasive surgery for colorectal cancer. JMA J 4(1):17–23. 10.31662/jmaj.2020-0089. (Epub 2021 Jan 14. PMID: 33575499; PMCID: PMC7872784) 33575499 10.31662/jmaj.2020-0089 PMC7872784

[4] Baek SJ, Piozzi GN, Kim SH (2021) Optimizing outcomes of colorectal cancer surgery with robotic platforms. Surg Oncol 37:101559. 10.1016/j.suronc.2021.101559 33839441 10.1016/j.suronc.2021.101559

[5] Melich G, Hong YK, Kim J, Hur H, Baik SH, Kim NK, Sender Liberman A, Min BS (2015) Simultaneous development of laparoscopy and robotics provides acceptable perioperative outcomes and shows robotics to have a faster learning curve and to be overall faster in rectal cancer surgery: analysis of novice MIS surgeon learning curves. Surg Endosc 29(3):558–568. 10.1007/s00464-014-3698-0 25030474 10.1007/s00464-014-3698-0

[6] de Azevedo JM, Vailati BB, Julião GPS, Fernandez LM, Perez RO (2019) Current surgical strategies in the management of rectal cancer. Curr Colorectal Cancer Rep 15(1):18–27. 10.1007/s11888-019-00428-0

[7] Miskovic D, Ahmed J, Bissett-Amess R, Gómez Ruiz M, Luca F, Jayne D, Figueiredo N, Heald RJ, Spinoglio G, Parvaiz A, European Academy for Robotic Colorectal Surgery (EARCS) (2019) European consensus on the standardization of robotic total mesorectal excision for rectal cancer. Colorectal Dis Off J Assoc Coloproctol Great Britain Ireland 21(3):270–276. 10.1111/codi.14502 10.1111/codi.14502 30489676

[8] Panteleimonitis S, Popeskou S, Aradaib M, Harper M, Ahmed J, Ahmad M, Qureshi T, Figueiredo N, Parvaiz A (2018) Implementation of robotic rectal surgery training programme: importance of standardisation and structured training. Langenbecks Arch Surg 403(6):749–760. 10.1007/s00423-018-1690-1. (Epub 2018 Jun 20. PMID: 29926187; PMCID: PMC6153605) 29926187 10.1007/s00423-018-1690-1 PMC6153605

[9] Feng Q, Yuan W, Li T, Tang B, Jia B, Zhou Y, Zhang W, Zhao R, Zhang C, Cheng L, Zhang X, Liang F, He G, Wei Y, Xu J, REAL Study Group (2022) Robotic versus laparoscopic surgery for middle and low rectal cancer (REAL): short-term outcomes of a multicentre randomised controlled trial. Lancet Gastroenterol Hepatol 7(11):991–1004. 10.1016/S2468-1253(22)00248-5 36087608 10.1016/S2468-1253(22)00248-5

[10] Jayne D, Pigazzi A, Marshall H, Croft J, Corrigan N, Copeland J, Quirke P, West N, Rautio T, Thomassen N, Tilney H, Gudgeon M, Bianchi PP, Edlin R, Hulme C, Brown J (2017) Effect of robotic-assisted vs conventional laparoscopic surgery on risk of conversion to open laparotomy among patients undergoing resection for rectal cancer: the ROLARR randomized clinical trial. JAMA 318(16):1569–1580. 10.1001/jama.2017.7219 29067426 10.1001/jama.2017.7219 PMC5818805

[11] Hemandas A, Flashman KG, Farrow J, O’Leary DP, Parvaiz A (2011) Modular training in laparoscopic colorectal surgery maximizes training opportunities without clinical compromise. World J Surg 35(2):409–414. 10.1007/s00268-010-0837-1. (PMID: 21052997) 21052997 10.1007/s00268-010-0837-1

[12] Quirke P, Steele R, Monson J, Grieve R, Khanna S, Couture J, O’Callaghan C, Myint AS, Bessell E, Thompson LC, Parmar M, Stephens RJ, Sebag-Montefiore D, MRC CR07/NCIC-CTG CO16 Trial Investigators, NCRI Colorectal Cancer Study Group (2009) Effect of the plane of surgery achieved on local recurrence in patients with operable rectal cancer: a prospective study using data from the MRC CR07 and NCIC-CTG CO16 randomised clinical trial. Lancet (Lond, Engl) 373(9666):821–828. 10.1016/S0140-6736(09)60485-2 10.1016/S0140-6736(09)60485-2 PMC2668948 19269520

[13] Fleshman J, Branda M, Sargent DJ, Boller AM, George V, Abbas M, Peters WR Jr, Maun D, Chang G, Herline A, Fichera A, Mutch M, Wexner S, Whiteford M, Marks J, Birnbaum E, Margolin D, Larson D, Marcello P, Posner M, Read T, Monson J, Wren SM, Pisters PW, Nelson H (2015) Effect of laparoscopic-assisted resection vs open resection of stage II or III rectal cancer on pathologic outcomes: the ACOSOG Z6051 randomized clinical trial. JAMA 314(13):1346–1355. 10.1001/jama.2015.10529. (PMID: 26441179; PMCID: PMC5140087) 26441179 10.1001/jama.2015.10529 PMC5140087

[14] Bonjer HJ, Deijen CL, Abis GA, Cuesta MA, van der Pas MH, de Lange-de Klerk ES, Lacy AM, Bemelman WA, Andersson J, Angenete E, Rosenberg J, Fuerst A, Haglind E, COLOR II Study Group (2015) A randomized trial of laparoscopic versus open surgery for rectal cancer. New Engl J Med 372(14):1324–1332. 10.1056/NEJMoa1414882 25830422 10.1056/NEJMoa1414882

[15] Kang SB, Park JW, Jeong SY et al (2010) Open versus laparoscopic surgery for mid or low rectal cancer after neoadjuvant chemoradiotherapy (COREAN trial): short-term outcomes of an open-label randomised controlled trial. Lancet Oncol 11(7):637–645 20610322 10.1016/S1470-2045(10)70131-5

[16] Lacy AM, Garcia-Valdecasas JC, Delgado S et al (2002) Laparoscopy-assisted colectomy versus open colectomy for treatment of non-metastatic colon cancer: a randomized trial. Lancet 359(9325):2224–2229 12103285 10.1016/S0140-6736(02)09290-5

[17] Weeks JC, Nelson H, Gelber S et al (2002) Clinical Outcomes of Surgical Therapy (COST) Study. Group short-term quality-of- life outcomes following laparoscopic-assisted colectomy vs open colectomy for colon cancer: a randomized trial. JAMA 287(3):321–328 11790211 10.1001/jama.287.3.321

[18] Guillou PJ, Quirke P, Thorpe H et al (2005) MRC CLASICC Trial Group. Short-term endpoints of conventional versus laparoscopic-assisted surgery in patients with colorectal cancer (MRC CLASICC trial): multicentre, randomised controlled trial. Lancet 365(9472):1718–1726 15894098 10.1016/S0140-6736(05)66545-2

[19] Stevenson AR, Solomon MJ, Lumley JW, Hewett P, Clouston AD, Gebski VJ, Davies L, Wilson K, Hague W, Simes J, ALaCaRT Investigators (2015) Effect of laparoscopic-assisted resection vs open resection on pathological outcomes in rectal cancer: the ALaCaRT randomized clinical trial. JAMA 314(13):1356–1363. 10.1001/jama.2015.12009 26441180 10.1001/jama.2015.12009

[20] Sciuto A, Merola G, De Palma GD, Sodo M, Pirozzi F, Bracale UM, Bracale U (2018) Predictive factors for anastomotic leakage after laparoscopic colorectal surgery. World J Gastroenterol 24(21):2247–2260. 10.3748/wjg.v24.i21.2247 29881234 10.3748/wjg.v24.i21.2247 PMC5989239

[21] Yamamoto S, Inomata M, Katayama H, Mizusawa J, Etoh T, Konishi F, Sugihara K, Watanabe M, Moriya Y, Kitano S, Japan Clinical Oncology Group Colorectal Cancer Study Group (2014) Short-term surgical outcomes from a randomized controlled trial to evaluate laparoscopic and open D3 dissection for stage II/III colon cancer: Japan Clinical Oncology Group Study JCOG 0404. Ann Surg 260(1):23–30. 10.1097/SLA.0000000000000499 24509190 10.1097/SLA.0000000000000499

